# The many connections of UFMylation with Alzheimer’s disease: a comprehensive review

**DOI:** 10.1186/s13024-025-00855-8

**Published:** 2025-06-04

**Authors:** Tingxiang Yan, Benjamin D. Clarkson, Zhenkun Lou, Wolfdieter Springer, Fabienne C. Fiesel

**Affiliations:** 1 Department of Neuroscience, Mayo Clinic, 4500 San Pablo Road, Jacksonville, FL 32224 USA; 2 Department of Laboratory Medicine and Pathology, Mayo Clinic, Rochester, MN 55905 USA; 3 Department of Oncology, Mayo Clinic, Rochester, MN 55905 USA; 4 Neuroscience PhD Program, Mayo Clinic Graduate School of Biomedical Sciences, Jacksonville, FL 32224 USA

## Abstract

Alzheimer’s disease (AD) is a complex neurodegenerative disorder that is characterized by the accumulation of pathologic tau and beta-amyloid proteins. UFMylation is an emerging ubiquitin-like post-translational modification that is crucial for healthy brain development. The UFM1 cascade was recently identified as a major modifier of tau aggregation in *vitro* and in *vivo*. Moreover, post-mortem AD brain shows pronounced alterations of UFMylation that are significantly associated with pathological tau, suggesting UFM1 might indeed be a modifier of human disease. However, the link between AD and UFMylation is yet to be fully explored. Interestingly, the UFMylation cascade is known to play important roles for several pathways that are known to be altered in AD, such as the DNA damage response, ER homeostasis, autophagy and the immune response. This review discusses the many connections between UFMylation with AD pathogenesis, emphasizing the role of UFMylation in these pathways and their abnormalities in AD. Understanding these connections is important to elucidate molecular mechanisms how UFM1 may impact AD and to uncover novel therapeutic strategies targeting UFMylation pathways for disease modification.

## Introduction

Alzheimer’s disease (AD) is a devastating neurodegenerative disorder that affects millions of people worldwide, causing progressive cognitive decline and memory loss [1, 2]. Despite extensive research, the underlying mechanisms of AD are not yet fully understood, and effective therapies remain elusive. Pathologic tau and amyloid-β (Aβ) deposits are the main neuropathological hallmarks of AD [3]. Their accumulation has been associated with abnormalities in various cellular processes, including DNA damage response (DDR) [4–7], endoplasmic reticulum (ER) homeostasis [8–11], autophagy functions [12–15], as well as the immune response [16–19]. These processes are critical for maintaining neuronal health, and their dysregulation has significant implications for the development and progression of AD.

Interestingly, many studies have highlighted the importance of UFMylation in these processes [20–24]. UFMylation is a ubiquitin-like (UBL) posttranslational modification that was discovered 20 years ago [24], but remains understudied. The UFM1 cascade is essential for proper brain development, as evidenced by the observation that complete loss of function in any component of the UFMylation pathway results in severe neurodevelopmental disorders [25–29]. Given this essential role of UFMylation for brain, this pathway may also play a significant role in the pathogenesis of age-related neurodegenerative diseases, such as AD. However, the role of UFMylation in AD is only beginning to emerge and remains to be fully explored.

**Fig. 1:**
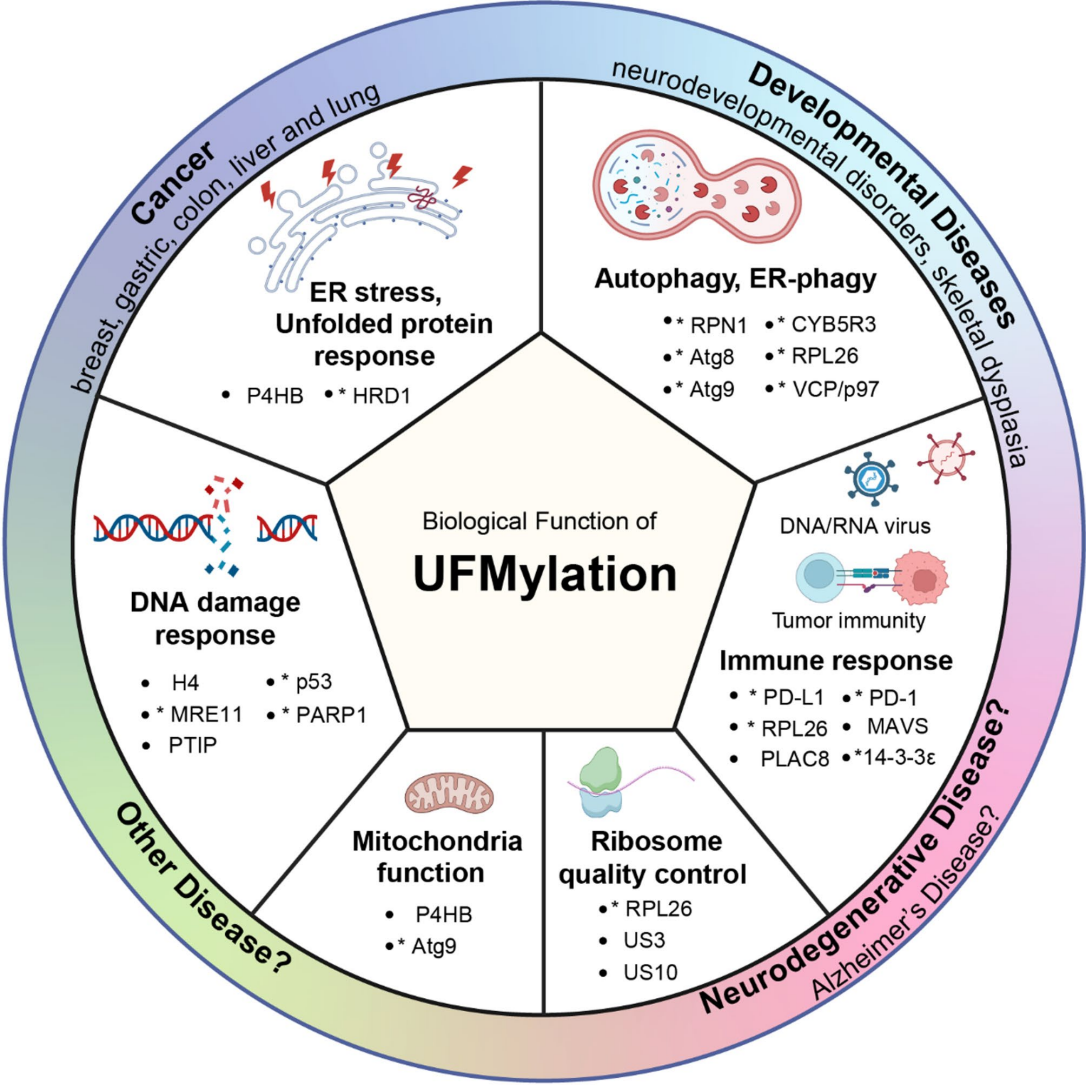
Key reported biological functions of UFMylation. The UFMylation pathway is known to play a role in various physiological processes including DNA damage response (DDR), endoplasmic reticulum (ER) stress response, regulating autophagy functions, and influencing the immune response. There is also evidence suggesting its involvement in mitochondrial and ribosome-associated protein quality controls. Dysregulation of UFMylation has been associated with developmental disorders, particularly neurodevelopmental disorders, various types of cancers, and it may also have a potential link with neurodegenerative diseases like Alzheimer’s Disease (AD). Reported proteins modified by UFM1 have been categorized based on the pathways they are involved in. Proteins labeled with “*” have been connected with AD

In this review, we provide an overview of the current understanding of UFMylation and its role in brain development and neurodevelopmental disorders, and we summarize the latest findings with regards to neurodegenerative disease. Given the strong overlap between pathways with UFM1 function and abnormities in AD, we further discuss common specific cellular processes such as the DDR, ER homeostasis, autophagy, and the immune response. We further propose future research directions to advance our understanding of the potential link between UFMylation and AD and discuss potential therapeutic strategies targeting UFMylation for the treatment of AD (Fig. 1).

### The ufmylation pathway

**Fig. 2:**
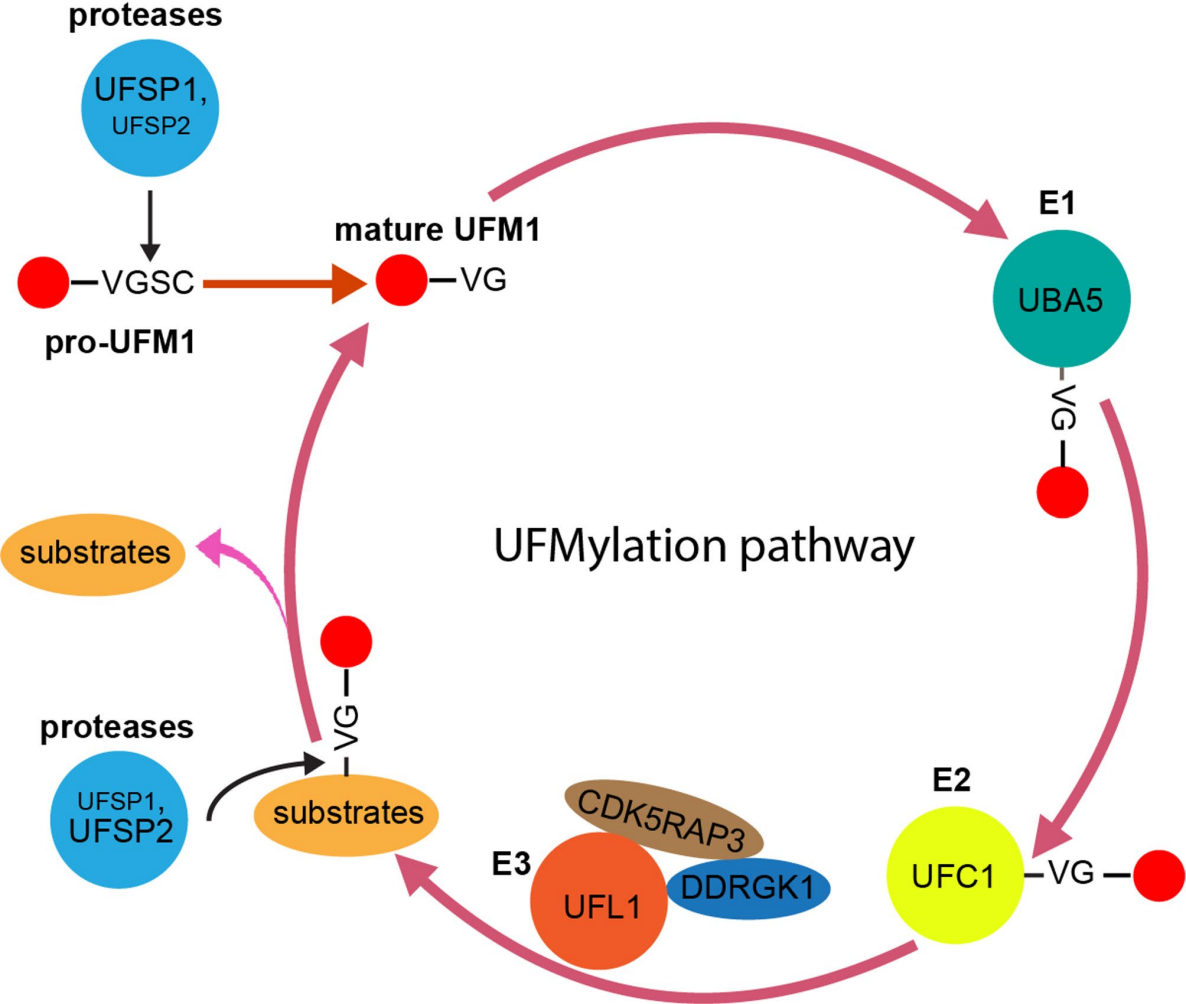
Cascade of the UFMylation and deUFMylation processes. The UFMylation pathway initiates with the precursor of UFM1 (pro-UFM). In the first step, UFM1-specific cysteine proteases, UFSP1 and UFSP2, enzymes cleave two amino acids from pro-UFM, resulting in mature UFM1 that exposes a glycine residue. Subsequently, UBA5 activates this mature UFM1. Once activated, UFM1 is handed over to UFC1. The critical step of ligating UFM1 to its target proteins is conducted by UFL1, aided by two adaptor proteins, DDRGK1 and CDK5RAP3, which expand the range of potential substrates for UFL1. The UFMylation process is reversible, with UFSP1/2 cleaving UFM1 from its substrates for recycling. Abbreviations: UFC1, ubiquitin fold-modifier conjugating enzyme 1; UFL1, ubiquitin fold-modifier specific ligase 1; UFM1, ubiquitin-fold modifier 1; VGSC: valine–glycine–serine–cysteine amino acids

Ubiquitin fold modifier 1 (UFM1) is a compact protein with 85 amino acids, and only a 15% sequence homology to ubiquitin [24, 30]. Its three-dimensional structure, however, which is characterized by a beta-grasp fold, is reminiscent of ubiquitin [30]. The process by which UFM1 is conjugated to its target proteins, termed UFMylation, follows a cascade involving a UFM1-specific set of E1, E2, and E3 enzymes, in analogy to the ubiquitin system [24, 31, 32] (Fig. 2). The initiation of this process necessitates the maturation of the precursor form of UFM1, known as proUFM1. This maturation is mediated by specific cysteine proteases, UFM1-specific peptidase UFSP1 and UFSP2, which trim the C-terminal Ser-Cys dipeptide of proUFM1 [33–35]. This reveals a single glycine residue, a departure from the typical diglycine C-terminus observed in other ubiquitin-like proteins (UBLs) [33].

Following its maturation, UFM1 undergoes activation via the pathway’s E1 enzyme, specifically referred to as ubiquitin activating enzyme 5 (UBA5) [24]. This activation occurs through an ATP-dependent mechanism, where UBA5 forms a high-energy thioester bond with the C-terminal glycine residue of UFM1 [36]. Next, UFM1 is transferred to the E2 conjugating enzyme in the cascade, Ubiquitin-fold modifier-conjugating enzyme 1 (UFC1) [24]. UFC1 also forms a thioester bond with UFM1, effectively accepting the activated UFM1 from UBA5 [37, 38]. This transfer is crucial for the subsequent ligation of UFM1 to its target protein, which is mediated by the E3 ligase in the cascade, UFM1-ligase 1 (UFL1) [31]. UFL1 plays a central role in determining the substrate specificity of the UFM1 conjugation system. It recognizes and binds to specific target proteins and facilitates the transfer of UFM1 from UFC1 to the target protein’s lysine residue [32]. This transfer results in the formation of an isopeptide bond between UFM1 and the target protein, effectively modifying the target protein and altering its function or stability. The exact molecular mechanisms and determinants of substrate recognition by UFL1 are not yet fully understood, and further research is needed to elucidate the principles governing substrate specificity in the UFM1 conjugation system [22, 25]. DDRGK1/UFBP1 and CDK5RAP3 are important components of the UFM1 conjugation system. DDRGK1 has been shown to form a large protein complex with UFL1, UFM1, and CDK5RAP3, suggesting a coordinated and spatially regulated role of these proteins in the UFM1 conjugation system [39]. DDRGK1 is mainly localized at the ER, and this might explain the outsized role of UFMylation for ER homeostasis detailed below [40].

The removal of UFM1 from target proteins is an essential process that allows for the recycling of UFM1 and the reversal of UFMylation-mediated protein modification. The deconjugation is mediated by the same two UFM1-specific proteases that mediate the maturation of UFM1 [33]. UFSP1 and UFSP2 recognize and cleave the isopeptide bond between UFM1 and the target protein, effectively releasing UFM1 and reverting the target protein back to its unmodified state. It is not clear whether UFSP1 and UFSP2 have entirely redundant functions. At least two studies have demonstrated that the deconjugation seems more reliant on UFSP2 compared to UFSP1. Knockdown of UFPS2 but not UFSP1 results in a significant increase in UFM1 conjugates [34, 35]. However, this could be cell-type specific. The dynamic process of conjugation and deconjugation is critical for maintaining cellular homeostasis and ensuring proper regulation of various cellular processes.

The balance between UFM1 conjugation and deconjugation is tightly regulated by multiple factors, including the availability of ATP, the expression levels of UFM1 system components, and the presence of cellular stress signals. Disruptions in this balance can lead to dysregulated protein function and have been implicated in various pathophysiological conditions [20–22, 25, 32] (Fig. 1). A better understanding of the molecular mechanisms governing the UFM1 conjugation and deconjugation processes, as well as the identification of novel target proteins and regulatory pathways, will be crucial for elucidating the biological functions of the UFM1 cascade in the brain under physiological and pathological conditions.

### The UFM1 cascade regulates neurodevelopment

UFMylation pathway genes and proteins are widely expressed across various human tissues, as indicated by The Human Protein Atlas (https://www.proteinatlas.org/). This is consistent with a significant role of the UFMylation pathway for the development of mice, as several systems including the hematopoietic, liver, intestinal, skeletal, cardiac, renal, immune, and central nervous systems, have been shown to be affected upon knockout (KO) of UFM1 pathway components [25, 27, 41–46]. In humans, several genetic variants in the genes encoding the core components of the UFMylation pathway have been associated with severe neurological and bone developmental disorders [23, 25] (Table 1), indicating that the UFM1 cascade is especially important for these processes. However, it remains unclear in which cell types and brain regions the UFM1 protein is expressed and how this is regulated.

**Table 1:** Mutations of the ufmylation machinery in human congenital disorders

Gene	SNP	sites of mutation	Mutation type	protein change	Biochemical/cellular impact	Disease/disorder and/or phenotype	Ref
UFM1	rs747359907	*NM_001286704.1: c.-273_-271delTCA*	Homozygous	273 − 271 del TCA (promoter)	Reduced gene expression in neuroblastoma and astroglioma cell lines	Hypomyelinating leukodystrophy type 14, global developmental delay, microcephaly, refractive epilepsy, and hypomyelination	[47]
	rs1033946108	*NM_016617.3: c.241 C > T*	Homozygous, hypomorphic	Arg81-to-Cys (R81C)	Reduced UFM1-UBA5 and UFM1-UFC1 intermediates formation	Early infantile encephalopathy, microcephaly, axial hypotonia, appendicular hypertonia, and epilepsy	[26]
UBA5	rs114925667	*NM_024818.3: c.1111G > A*	Biallelic Variants	Ala371-to-Thr (A371T)	Reduced UFM1-conjugates formation	Early-onset encephalopathy, intellectual deficiency, microcephaly and early-onset intractable epilepsy	[27, 48]
	rs886039756	*NM_024818.3: c.181 C-T*	Biallelic Variants	Arg61-to-Ter (R61X)	N/A		
	rs374052333	*NM_024818.6: c.562 C > T*	Biallelic Variants	Arg188-to-Ter (R188X)	N/A		
	rs886039757	*NM_024818.3: c.904 C > T*	Biallelic Variants	Gln302-to-Ter (Q302X)	Reduced UBA5-UFM1 levels		
	rs886039758	*NM_024818.3: c.971_972insC*	Biallelic Variants	frameshift and termination	Reduced thioester formation with UFM1; reduced transfer of UFM1 to UFC1		
	rs886039759	*NM_024818.3: c.778G > A*	Biallelic Variants	Val260-to-Met (V260M)	Reduced thioester formation with UFM1; reduced transfer of UFM1 to UFC1		
	rs886039760	*NM_024818.3: c.1165G > T*	Biallelic Variants	Asp389-to-Tyr (D389Y)	Slightly reduced UFM1-conjugates formation		
	rs532178791	*NM_024818.3: c.169 A > G*	Biallelic Variants	Met57-to-Val (M57V)	Reduced thioester formation with UFM1; reduced transfer of UFM1 to UFC1		
	rs886039761	*NM_024818.3: c.503G > A*	Biallelic Variants	Gly168-to-Glu (G168E)	Reduced UFM1-conjugates formation		
	rs774318611	*NM_024818.3: c.164G > A*	Biallelic Variants	Arg55-to-His (R55H)	Reduced UFM1-conjugates formation		
	rs1553770577	*NM_024818.4: c.907T > C*	N/A	Cys303-to-Arg (C303R)	N/A		
	rs745968949	*NM_024818.3: c.855 C-A*	Biallelic Variants	Tyr285-to-Ter (Y285X)	Reduced mRNA and protein level		
	N/A	*NM_024818.4: c.761T > C*	Biallelic Variants	Leu254-to-Pro (L254P)	Impaired UBA5-UFM1 interaction; reduced transfer of UFM1 to UFC1		
	rs540839115	*NM_198329.2: c.568 C > T*	Biallelic Variants	Arg246-to-Ter (R246X)	Increased nuclear localization, decreased protein half-life, reduced thioester formation with UFM1	Autosomal recessive cerebellar ataxia	[49]
	rs886039762	*NM_198329.2: c.760 A > G*	Biallelic Variants	Lys310-to-Glu (K310E)	Slightly decreased protein half-life		[49]
	rs767107499	*NM_024818.4: c.895 C > T*	Homozygous	Pro299-to-Ser (P299S)	Impaired UBA5-UFM1 interaction; reduced transfer of UFM1 to UFC1	Global developmental delay, epilepsy and hypomyelination	[51]
	N/A	*NM_024818.4: c.158 A > T*	Homozygous	Tyr53-to-Phe (Y53F)	Impaired UBA5-UFM1 interaction; reduced transfer of UFM1 to UFC1	Early myoclonic epilepsy	[52]
	rs771295288	*NM_024818.3: c.31 C > T*	Homozygous	Arg11-to-Trp (R11W)	Reduced levels of UBA5 protein; Impaired UBA5-UFM1 interaction	Severe congenital neuropathy	[53]
UFC1	rs1181612302	*NM_016406.3:c.68G > A*	Homozygous, hypomorphic:	Arg23-to-Gln (R23Q)	reduced UFM1-UFC1 intermediate and UFM1 conjugates	Neurodevelopmental disorder with spasticity and poor growth	[26]
	rs1553232770	*NM_016406.3: c.317 C > T*	Homozygous, hypomorphic:	Thr106-to-Ile (T106I)	reduced UFM1-UFC1 intermediate and UFM1 conjugates		[26]
UFSP2	N/A	*NM_018359.3: c.1373 A > G*	N/A	Tyr458-to-Cys (Y458C)	N/A	Cerebral visual impairment	[54]
	rs142500730	*NM_018359.3: c.344T > A*	Homozygous	Val115-to-Glu (V115E)	reduced UFSP2 protein level	Developmental and epileptic encephalopathy	[28]
	rs796052130*	*NM_138668.2: c.868T > C*	Heterozygous	Tyr290-to-His (Y290H)	impaired deUFMylation activity	Beukes hip dysplasia	[281]
	rs1554022725*	*NM_018359.3: c.1277 A > C*	Heterozygous	Asp426-to-Ala (D426A)	reduced deUFMylation activity	Spondyloepimetaphyseal dysplasia, Di Rocco type	[282]
	rs2095515802*	*NM_018359: c.1283 A-G*	Heterozygous	His428-to-Arg (H428R)	reduced deUFMylation activity		[283]
DDRGK1	rs1325869434*	*NM_023935.3:c.408 + 1G > A*	Homozygous	IVS3DS, G-A, +1	no protein expression	Spondyloepimetaphyseal dysplasia, Shohat type	[45]

*these mutations are not linked to neurodevelopmental diseases, but have been added for completion of all UFM1-related variants linked to human congenital diseases

A three base pair homozygous deletion in the promoter region of the UFM1 gene has been identified in patients exhibiting hypomyelination and atrophy of the basal ganglia and cerebellum. It was shown that this mutation results in reduced UFM1 expression in neuroblastoma (SH-SY5Y) and astroglioma (U371) cell lines, but not in other tested cell lines (HeLa (cervical adenocarcinoma) or HOG-F2 (oligodendroglioma)) [47]. Moreover, a homozygous missense mutation in UFM1 (UFM1 p.R81C) has been observed in patients suffering from severe early-onset encephalopathy accompanied by progressive microcephaly. This specific missense mutation significantly hinders the formation of both UFM1-UBA5 and UFM1-UFC1 intermediates, leading to impaired UFMylation of cellular proteins [26]. Furthermore, eighteen UBA5 variants have been detected in patients with neurodevelopmental disorders such as hypomyelination with atrophy, early-onset encephalopathy, early myoclonic epilepsy and severe infantile-onset encephalopathy [27, 48–53]. Most UBA5 variants result in a loss of function by reducing UBA5 expression levels, decreasing protein half-life, attenuating UFM1 activation, or impairing the transfer of activated UFM1 to UFC1. Notably, the UBA5 (Arg246-to-Ter (R246X)) mutation exhibits increased nuclear localization; however, it also demonstrates loss of function due to reduced protein half-life and diminished UFM1 activation (Table 1). In addition, two UFC1 mutations have been found in patients with severe early-onset encephalopathy and progressive microcephaly [26]. These UFC1 mutations adversely affect the formation of UFM1-UFC1 intermediates, resulting in a widespread reduction in protein UFMylation. UFSP2 mutations have also been discovered in patients presenting with cerebral visual impairment [54]. In a recent study, a UFSP2 variant has been reported as the causative mutation for an autosomal recessive form of pediatric neurodevelopmental anomalies and epilepsy [28]. This UFSP2 mutation reduced the abundance of UFPS2 which was concomitant with an increased abundance of UFMylated targets, indicating the variant may impair de-UFMylation rather than UFMylation. The potential connection between neurodevelopmental and neurodegenerative diseases has garnered significant attention in recent research [55–59]. However, while the essential role of UFMylation for neurodevelopment disorders is evident, its role for degenerative diseases, such as AD, is just emerging.

### UFM1 is a novel regulator of Tau aggregation and emerging AD modifier

Recently, the UFMylation cascade was identified as a novel key modifier of tau aggregation. Samelson et al.. conducted a genome-wide CRISPRi-based modifier screen in induced pluripotent stem cell (iPSC)-derived neurons to systematically identify cellular factors involved in the accumulation of tau aggregates in human neurons [60]. They discovered that repression of genes essential for UFMylation, such as UFM1, UFL1, and DDRGK1, led to a reduction in tau oligomer levels. Based on this, Parra Bravo et al. tested a subset of genes using a newly developed iPSC-derived 4R tauopathy model. This confirmed the UFMylation cascade as a major positive regulator of tau propagation. The authors further demonstrated that inhibition of the UFMylation pathway by knocking down the E1 enzyme UBA5 reduced tau spreading in the PS19 tau mouse model [61]. Together this data suggests that the UFM1 cascade is a powerful regulator of tau aggregation and spreading in vitro and in vivo. The mechanism of this regulation remains unknown. It had been speculated that the reduction of UFMylation might involve the reduction of endogenous tau protein [61], although the effect size on endogenous tau protein levels was much smaller compared to the effect on tau aggregation [60].

To investigate the role of the UFMylation pathway for human disease, we meta-analyzed a single-cell nuclear transcriptomic dataset of normal and AD brains [62, 63]. We assessed the expression of UFMylation pathway components across six primary cell types and found specific changes in excitatory neurons: five out of eight UFMylation pathway components (UFSP1, UFSP2, UFC1, UFL1, and DDRGK1) showed significant reduction in AD compared to controls [63]. Given the vulnerability of excitatory neurons to neurodegeneration, these results suggest that disruptions in the UFMylation pathway may play a role in the pathogenesis of AD. In fact, using a biochemical analysis of frozen brain tissue, we further discovered profound changes in UFSP2 and UFM1 protein levels in the frontal and temporal AD cortex of individuals with AD [63]. We found that conjugated UFM1 levels were increased in AD, while soluble UFSP2 levels were decreased in these affected regions but were not changed in the unaffected cerebellum. Importantly, in human brain UFM1 levels positively correlated with pathological tau levels (including insoluble total tau, soluble phosphorylated tau (p-tau), and insoluble p-tau) but not with total tau in the temporal and frontal cortex [63], supporting a role of UFM1 as tau aggregation modifier in AD.

Based on these findings, the UFM1 cascade seems an attractive pathway to target tau aggregation. However, given the unknown mechanism of how UFM regulates tau and which substrates it is conjugated to in AD brain, more work is needed validate the UFM1 pathway as a potential therapeutic avenue. Moreover, given the broad roles of UFM1 in a variety of stress-related pathways, abnormal UFMylation might have far-reaching and unwanted consequences. As detailed below there are a multitude of pathways known to be influenced by UFM1 that play a role for AD pathogenesis. It will be important to validate these as potential candidate pathways in order to further develop UFMylation modifying treatments into a viable therapeutic strategy.

## Functional pathways that connect UFMylation and AD

### The DNA damage response (DDR)

#### Overview and role in AD

DNA damage, an alteration in the chemical structure of DNA, is a key feature in post-mitotic neurons, which rely heavily on robust repair mechanisms to maintain genomic stability [64]. Neuronal DNA undergoes continuous, non-random breakage and repair. Recent studies mapped sites of DNA repair synthesis by sequencing in post-mitotic neurons have identified repair hotspots across the genome [65, 66]. These hotspots are enriched with histone H2A isoforms, RNA-binding proteins, and evolutionarily conserved elements of the human genome [66]. Notably, they are predominantly linked to single-strand breaks at CpG-methylated neuronal enhancers [65]. Among various types of DNA damage, double-strand breaks (DSBs) are most lethal. DSBs are primarily repaired through homologous recombination (HR) and non-homologous end-joining (NHEJ) [67, 68]. HR, an error-free pathway active in the post-S phase, uses sister chromatids and proteins like CtIP and RAD51, with DNA synthesis by polymerases. In contrast, NHEJ is error-prone but operates throughout the cell cycle, utilizing KU70/80, DNA-PK, and ligation by LIG4, XRCC4, and XLF. In neurons, NHEJ is the dominant repair pathway due to their post-mitotic state, but HR also plays a significant role [69]. The MRN complex (MRE11, RAD50, and NBS1) is crucial for HR, recruiting repair proteins and being activated by ATM kinase, while also supporting NHEJ [70–73]. The choice between HR and NHEJ depends on factors like DNA damage type, cell cycle phase, and competitive binding at the DSB site by BRCA1 (favoring HR) and 53BP1 (favoring NHEJ) [74, 75].

DNA damage and impaired DNA repair are key characteristics of aging and various neurodegenerative disorders, including AD [76–82]. Studies on postmortem human brain samples have demonstrated a significant increase in γH2AX, a widely accepted marker of DSBs [83], in neurons and astrocytes of AD brain, indicating elevated DNA damage in these cells [7, 80, 84]. Additionally, high γH2AX signals have been detected in oligodendrocytes within the white matter of AD brains [6, 85]. Although these γH2AX signals were not significantly different between controls and AD, they were markedly elevated in other dementias [85], and oxidative DNA damage in oligodendrocytes has been identified as a characteristic feature of white matter lesion pathogenesis in the aging human brain [86]. The accumulation of DSBs in AD is largely attributed to increased oxidative stress, driven by the pathogenesis of Aβ and tau [87–90]. In line with the accumulation of γH2AX signal, increased levels of the other DNA damage sensors such as 53BP1, and phospho-Ser1981 ATM (p-ATM) have also been reported [6, 79, 80, 91], although total levels of ATM and MRN proteins seem reduced in AD cortical neurons, which could further contribute to the accumulation of DNA damage [92, 93]. Studies with both human AD samples and animal models further indicate that the NHEJ DNA repair pathway is compromised in AD [4, 94]. The compromised ability to repair DSBs in AD has been suggested to cause genomic instability, tau hyperphosphorylation, and apoptosis, all of which exacerbate neurodegeneration [6, 76, 89, 95].

#### The role of ufmylation and connections with AD

UFMylation plays a crucial role in the DDR. Loss of UFL1 increases DDR in the absence of exogenous stress, leads to p53 activation, and enhanced cell death [46]. The formation of the MRN complex, its immediate recruitment to DNA damage sites, and proper ATM activation for initiating DNA repair all rely on UFL1-mediated UFMylation of MRE11 on lysine 282 [96]. ATM phosphorylates UFL1 before the activation and recruitment of the MRN complex to DSBs, thereby enhancing the ligase activity of UFL1 and leading to the UFMylation of histone H4 in a Tip60-dependent manner. This creates a positive feedback loop for ATM activation [97]. This process is counteracted by ATM induced de-UFMylation. Upon ATM-mediated phosphorylation, UFSP2 relocates from the MRN complex to double-strand breaks, where it de-UFMylates H4 [98]. Interestingly, our work showed that knocking out UFSP2 enhanced survival against DNA damage in a UFM1-dependent mechanism in neural progenitors that underwent neuronal differentiation, but not in naive progenitors [63]. The reason for this difference remains unclear and needs to be explored further. The UFMylation cascade has also been implicated in the stabilization of the replication fork. In BRCA1/2-deficient cells, UFL1 localizes to stalled replication forks and facilitates the UFMylation of PTIP, a component of the MLL3/4 methyltransferase complex [99]. This modification promotes the assembly of the PTIP-MLL3/4 complex, leading to increased H3K4me1 and H3K4me3 levels at stalled replication forks and the subsequent recruitment of the MRE11 nuclease.

In addition to MRE11, two other of the five reported UFM1-modified DDR substrates, p53 and poly (ADP-ribose) polymerase-1 (PARP1), have been linked to AD. UFMylation of the tumor suppressor p53, particularly at four lysine residues in its C-terminal domain, is crucial for its stabilization and enhanced transcriptional activity. This promotes genomic integrity and suppresses tumorigenesis. UFMylation of p53 impedes MDM2 ligase recruitment, reducing ubiquitylation and subsequent degradation of p53 [100]. PARP1, a key sensor of DNA damage and replication stress [101–103] known to play a critical role in genome integrity is also modified by UFM1 [104]. An in vitro study showed that PARP1 is UFMylated at K548, which enhances its catalytic activity. PARP UFMylation also supports CHK1 activation, which stabilizes replication forks under conditions of stress, thereby contributing to the preservation of genomic stability [104].

Both cell-based and animal studies suggest a protective role of normal tau protein in the repair of DSBs [6, 105–107]. Knockdown of endogenous tau in neurons leads to elevated levels of DNA damage, indicated by increased γH2AX [6]. Furthermore, neurons from tau KO mice show higher levels of DNA damage compared to WT neurons [107]. Tau translocates to the nucleus under heat or oxidative stress [106] and upon treatment with DNA damage-inducing agents such as etoposide [6]. The phosphorylation of tau that has been observed under such conditions might be facilitated by checkpoint kinases that are known to regulate DNA replication and the cell cycle in response to DNA damage [108]. However, pathological tau disrupts the neuronal DNA repair system, leading to the accumulation of DNA damage [88, 109]. In addition to exacerbating oxidative stress, abnormal tau interacts with and sequesters critical DNA repair proteins, hindering their ability to function effectively at sites of DNA damage [88, 110, 111]. In AD and other tauopathies, hyperphosphorylated and aggregated tau interferes with nuclear transport, mislocalizing critical repair proteins such as 53BP1 and BRCA1 [110]. This disruption may stem from tau’s interaction with nucleoporins like Nup98, which compromises nucleus-cytoplasm trafficking and contributes to tauopathy [111]. Moreover, tau oligomers bind p53 in the cytoplasm, impairing its nuclear relocation and DDR, ultimately leading to neuronal death [88]. Pathological tau also disrupts microtubule organization, further hindering the trafficking of DNA repair proteins [6]. These observations suggest a role of tau in DDR pathways and genomic stability. However, it is unclear whether this function of tau plays a major role in the pathogenesis of AD. Nevertheless, UFMylation induced tau-dependent or -independent effects on the DNA damage response might contribute to the genomic instability associated with AD.

Given the higher levels of conjugated UFM1 in AD brains [63], it is possible several substrates of UFM1 involved in the DDR are hyperUFMylated. Higher UFMylation of MRE11 might improve DNA damage repair while hyperUFMylation of p53 might contribute to the p53 accumulation that is observed in AD brain [112], which is further associated with p53 aggregation and excessive DNA damage [88]. Moreover, hyperUFMylation of PARP1 could enhance its activity, potentially contributing to the accumulation of ADP-ribose polymers and NAD + depletion observed in human AD brains [113, 114]. Additionally, the interplay between UFMylation and other cellular processes implicated in AD, such as autophagy and ER stress, may further impact the DDR. Further research is needed to elucidate the precise molecular mechanisms underlying the potential link between AD and UFMylation in the context of DDR and determine the therapeutic implications of targeting UFMylation to improve DDR in AD.

### ER stress response

#### Overview and role in AD

The ER is a critical cellular organelle involved in protein synthesis. When the protein-folding capacity of the ER is overwhelmed, the accumulation of misfolded or unfolded proteins leads to a condition known as ER stress. This triggers the activation of the unfolded protein response (UPR), a signaling pathway that increases the production of ER-resident chaperones and foldases to assist with protein folding [115, 116]. The UPR also reduces the synthesis of new and promotes the degradation of misfolded proteins. There are three main ER transmembrane sensors: inositol-requiring enzyme 1 (IRE1), protein kinase RNA-like ER kinase (PERK), and activating transcription factor 6 (ATF6), which are kept in an inactive state by binding to the ER chaperone glucose-regulated protein 78 (GRP78), also known as BiP [117]. Accumulation of misfolded proteins leads to a lower availability of GRP78 to bind and inhibit ER transmembrane sensors, leading to their activation.

ER stress and the UPR play a significant role in AD [8, 118–122]. The accumulation of misfolded tau and Aβ causes ER stress and leads to the activation of the UPR [9–11]. Chronic activation of the UPR, however, can result in sustained ER stress, which has been associated with the development and progression of AD [9, 118, 123]. It is interesting to note that IRE1 phosphorylation, triggered by ER stress, correlates well with Braak tau stages, which classify the progression of tau pathology in the brain [123]. Conditional deletion of the RNase domain of IRE1 in the nervous system significantly reduced amyloid load and soluble and insoluble Aβ species in an AD mouse model by regulating amyloid precursor protein (APP) degradation at the ER through IRE1/XBP1 signaling [123]. ER stress further accelerates pathological tau phosphorylation via PERK-eIF2α activation in rTg4510 mice and it was shown that treatment with a PERK inhibitor effectively reduces levels of phospho-tau [124]. Moreover, ER stress is connected with several other pathological features of AD, such as synaptic dysfunction and neuroinflammation [11, 125, 126], although the molecular mechanisms remain enigmatic. A deeper understanding of how ER homeostasis and the UPR relate to the pathogenesis of AD will offer insights into the molecular mechanisms of AD and might reveal new therapeutic targets.

#### The role of ufmylation and connections with AD

The UFM1 cascade plays an essential role in preserving ER balance and is involved in the UPR during ER stress [20–22, 25, 32, 127, 128]. Numerous studies have found a direct connection between UFMylation and ER stress, with the UFM1 system being transcriptionally upregulated in various situations such as ischemic heart disease, diabetic mouse macrophages, exocrine pancreas, gut inflammation and kidney atrophy [42, 44, 129–131] and upon ER stress induction in cells, potentially by being direct targets of the XBP1 transcription factor [132]. Knockdown of DDRGK1 increased cell death in a beta-cell line upon ER stress [39], increased ER stress and activation of the UPR along with the cell death program in intestinal epithelial cells [42]. Loss of UFL1 was linked to elevated ER stress and UPR induction in bone marrow cells and to kidney atrophy due to dysfunctional ER homeostasis [44]. Mechanistically, it has been shown that the depletion of DDRGK1 inhibits IRE1α-XBP1 signaling and activates the PERK-eIF2α-CHOP apoptotic pathway through targeting the ER stress sensor IRE1α [40], while UFL1 regulates PERK signaling to prevent cardiomyocyte cell death [43].

Prolyl 4-hydroxylase beta (P4HB), a chaperone that aids in correct disulfide bond formation during protein folding, plays a crucial role for ER stress [133]. UFMylation of P4HB at Lys69/114/130 is essential for maintaining its stability and the loss of UFMylation at these sites lead to its degradation via the ubiquitin-proteasome pathway, resulting in increased oxidative and ER stress [134]. The UFMylation of HRD1, an ER-associated protein degradation ubiquitin ligase, at Lys610, causes activation and leads to enhanced degradation of misfolded proteins, thereby maintaining ER homeostasis [135]. It has been suggested that HRD1 plays a key role in AD by targeting misfolded proteins for degradation [136]. HRD1 deficiency leads to the accumulation of APP, which is cleaved into Aβ [137]. Additionally, it has been shown that HRD1 targets tau and abnormal p-tau for proteasomal degradation [138]. Significantly reduced HRD1 protein levels have been identified in the cerebral cortex of AD patients [137], contributing to impaired protein degradation and the progression of AD pathology.

The association between the UFM1 system and ER stress is widespread and consistent phenomenon, with UFM1 modification potentially shielding cells from ER stress-induced apoptosis by preserving ER homeostasis. However, in our hands, KO of UFSP2 also caused the induction of the UPR under basal conditions and led to enhanced UPR and neuronal death upon ER stress [63], suggesting that both the reduction as well as the abnormal accumulation of UFMylated proteins can exacerbate the UPR. Whether more conjugated UFM1 as observed in AD also induces the UPR remains unknown. Investigating the molecular connections between UFMylation, ER homeostasis, and UPR in AD will help understand how the UPR and ER stress contribute to the pathogenesis of AD and might unveil potential therapeutic strategies to counteract the harmful effects of ER stress and UPR activation in the disease.

### Autophagy

#### Overview and role in AD

Autophagy serves as a crucial cellular process that maintains metabolic homeostasis by breaking down and recycling cellular components [139–141]. The mammalian target of rapamycin complex 1 (mTORC1) holds a pivotal role in the biogenesis of autophagy, as it regulates the process in response to nutritional conditions [142–144]. Neurons exhibit a high degree of polarization, with autophagosomes predominantly forming at axonal tips and subsequently maturing during retrograde transport to the cell soma [145–147]. The biogenesis of autophagosomes entails the recruitment of Atg13 and Atg5 to Double-FYVE Containing Protein 1 (DFCP-1), an ER structure enriched in Phosphatidylinositol 3-Phosphate (PI3P). Following this step, lipidated LC3 is incorporated into the developing autophagosomes [148]. Notably, autophagy induction occurs more efficiently in younger neurons as the expression of several essential autophagy genes declines with age [14, 146, 149, 150]. Reduction of autophagy has been suggested to contribute to various age-related neurodegenerative diseases, including AD, highlighting the importance of understanding the intricate mechanisms of autophagy in neuronal cells.

Dysregulation of autophagy in AD is evident in both animal models and human patients [14, 146, 151–155]. Dystrophic neurites, abnormal neuronal processes that are a common feature in AD brain, show a large number of immature autophagic vacuoles, suggesting widespread deficiency of autophagy in AD brain [12, 154, 156, 157]. Moreover, several autophagy-related proteins were found downregulated during the progression of AD [155, 158, 159]. Autophagy malfunction has been linked to Apolipoprotein E4 as well as APP, presenilin-1 (PS-1), and presenilin-2 (PS-2) and therefore seems to play a role for sporadic and familial forms of AD [12, 160–164]. However, the mechanism(s) of how these genes affect autophagy remains largely elusive. Biochemical analyses reveal a dramatic increase in Atg8/LC3 in postmortem AD brains, with co-localization to hyperphosphorylated tau, and a significant elevation in APP/PS1 mice [154, 165]. It is noteworthy that the central elements of AD pathology, Aβ and tau, both have a complex relationship with autophagy [15, 166, 167]. In the early stages of AD, the accumulation of Aβ activates autophagy as a compensatory mechanism to promote their clearance [168–171]. Autophagy also regulates APP metabolism by inhibiting its proteolytic processing and enhancing its degradation through Atg5-dependent pathways, thereby reducing Aβ production [172]. Additionally, autophagy plays a critical role in the secretion of both Aβ and tau. For instance, autophagy deficiency caused by Atg7 knockdown impairs the secretion of Aβ into the extracellular space [173], while disrupted autophagic flux, induced by p300/CBP hyperactivation or bafilomycin A1 treatment, has been shown to increase tau secretion in neurons [174]. As AD progresses, the continuous production and accumulation of pathological Aβ and tau lead to significant disruption of the autophagy–lysosomal pathway [173, 175–178]. This dysfunction causes further aggregation of Aβ and tau, which in turn exacerbates the impairment of the autophagy–lysosomal system. This vicious cycle ultimately drives the formation of Aβ plaques and neurofibrillary tangles, contributing to the neurodegeneration observed in AD. However, enhancing autophagy has been shown to reduce Aβ levels and facilitate the degradation of pathological tau [167, 178–180], highlighting its potential as a therapeutic target for AD.

#### The role of UFMylation and connections with AD

UFMylation has been identified as a novel regulator of SQSTM1/p62, an essential protein in autophagy that is decorated by ubiquitin as well as several other UBLs [181]. However, the effect of UFM1 on p62 seemed to occur indirectly, through an elevated cell type-specific ER stress response, which led to p62 accumulation in the cytosol and was not observed in all investigated cell types. It is interesting that p62 has been implicated in the degradation of tau protein [182, 183], but it remains unknown whether this or other mechanisms are associated with the regulation of tau by the UFM1 cascade. In bone marrow cells, upregulation of p62 was also observed upon loss of UFL1 but here, loss of UFMylation also caused an increase of LC3-II and blocked autophagic degradation [46]. In line with a role for autophagic degradation, Atg9, which plays a role in autophagosome expansion, was identified as a conserved target of UFM1. The UFMylation of Atg9 is essential for maintaining Atg9A protein levels to coordinate autophagy [184]. In both 5XFAD and APP/PS1 mouse models, Atg9A accumulates extensively in dystrophic neurites surrounding amyloid plaques [185], but it is unclear whether this could be related to hyperUFMylation. In addition to degradation, a role of UFMylation for autophagy initiation has been suggested via UFMylation of the VCP/p97 protein at lysine 109 (K109). This was reported to enhance the stabilization of BECN1 and the assembly of the PtdIns3K complex [186]. VCP has been shown to mediate disaggregation of ubiquitylated tau fibrils [187].

UFMylation further plays crucial role in the selective autophagy process known as ER-phagy [184, 188, 189]. This process is essential for maintaining ER homeostasis under various stress conditions, cellular development, and environmental challenges [190–194]. It was shown that UFM1 conjugates to specific ER proteins via DDRGK1 and the UFL1 ligase [188]. This facilitates the degradation of ER sheets similar to PINK1-PRKN induced mitophagy [195]. UFL1 is recruited to the ER by DDRGK1 and there conjugates UFM1 to the substrate ribophorin I (RPN1) and ribosomal protein L26 (RPL26), which facilitates ER-phagy [188]. Interestingly, RPN1 is elevated in brain capillaries of AD patients [196]. Furthermore, RPL26 immunoreactivity is reduced in AD neurons, and it has been identified as a potential biomarker for AD pathogenesis [197, 198]. UFMylation of NADH-cytochrome b5 reductase 3 (CYB5R3) inactivates this protein and also induces ER-phagy [189]. The degradation of UFMylated CYB5R3 in lysosomes depends on the autophagy-related protein Atg7 and the autophagy-adaptor protein CDK5RAP3, which possesses non-canonical shuffled Atg8 interacting motifs essential for Atg8 interaction and autophagy initiation [199]. A crucial role of ER-phagy for AD is not established. However, it has been found CYB5R3 levels are reduced in the cerebrospinal fluid of 5XFAD mice [200]. Together with the above-mentioned changes in RPN1 and RPL26, this suggests that ER-phagy might be dysregulated in AD. In line with this, a recent study showed impaired ER-phagy in a Drosophila model expressing human AAP. In this model, moderate upregulation of ER-phagy in the brain was sufficient to drive APP degradation and to significantly mitigate the associated phenotypes [201]. It is interesting that mutations in the RTN3 gene, an ER-phagy receptor, have been detected in several early and late onset AD cases [202]. While the pathogenicity remains to be determined, RTN3 appears to play a role in regulating the production of Aβ by inhibiting BACE1 activity to produce amyloid beta-protein [203, 204]. These findings highlight the potential of targeting ER-phagy as a therapeutic strategy for treating diseases linked to ER protein aggregation.

In summary, UFMylation plays a multifaceted role in autophagy regulation, specifically ER-phagy, by modulating key proteins and pathways essential for maintaining cellular homeostasis, development, and stress response. While dysfunction of autophagy has been widely reported in AD, it is noteworthy that all identified autophagy-related UFM1 substrates—VCP, RPN1, CYB5R3, Atg8/LC3, Atg9A, and RPL26—have demonstrated links to AD. These findings highlight the significance of UFMylation in regulating autophagy across diverse biological contexts and organisms. Investigating the precise molecular mechanisms through which UFMylation might influence autophagy dysfunction in AD could offer insights into the disease’s pathogenesis and highlight novel therapeutic targets.

### Immune response

#### Overview and role in AD

An immune response is fundamentally defined as the body’s reaction to foreign substances [205]. Within the central nervous system, this immune response is termed neuroinflammation, reflecting a mechanism vital for maintaining homeostasis and guarding against pathogenic insults [16]. Neuroinflammation can be triggered by a variety of pathological conditions, including infections, traumatic injuries, ischemic events, or exposure to toxins and is mostly mediated by microglia and astrocytes although other cell types also participate depending on the context [16, 19, 206]. The resulting cascade involves the secretion of pro-inflammatory cytokines such as interleukins, tumor necrosis factor (TNF), and chemokines.

Abnormal neuroinflammation is one of the major hallmarks of AD and linked to disease pathogenesis. Chronic activation of microglia and astrocytes fosters a persistent inflammatory state marked by excessive production of cytokines, synaptic pruning, and neuronal damage [16, 207–210]. This maladaptive immune response is further exacerbated by dysregulated transcription factors such as toll-like receptor 4 (TLR4)/nuclear factor kappa B (NF-κB), which amplify pro-inflammatory signaling pathways, and the activation of inflammasomes, including NLRP3, which drive excessive inflammatory cytokine release [211–214]. Immune checkpoints, such as Programmed death receptor 1/programmed death receptor-ligand 1 (PD-1/PD-L1), which normally maintain immune balance by modulating microglial activation and limiting excessive inflammation, also become dysfunctional in AD, further perpetuating neuroinflammation [215, 216]. Additionally, viral infections, including those caused by herpes simplex virus (HSV-1), Hepatitis C Virus (HCV) and Severe Acute Respiratory Syndrome Coronavirus 2 (SARS-CoV-2), have been linked to the activation of innate immune responses, contributing to the inflammatory burden in AD [217–219]. Together, these elements contribute to a self-perpetuating cycle of neuroinflammation and neurodegeneration in AD, underscoring the potential therapeutic value of targeting immune pathways. Given the strong neuroinflammatory phenotypes in AD, it is not surprising that many AD-associated risk genes identified through genome-wide association studies, e.g. CD33, INPP5D, CLU, CR1, SPI1, ABCA7, EPHA1, the MS4A gene cluster, and TREM2 have been implicated in the adaptive and innate immune systems [220–223].

#### The role of UFMylation and connections with AD

Neuron-specific deletion of UFL1 or DDRGK1 both lead to elevated inflammatory responses in mouse brain [224], indicating that the UFM1 cascade is very important for the immune response in the nervous system. Consistently, recent studies have demonstrated the involvement of UFMylation in several aspects of the immune response, including the regulation of transcription factors, inflammasome, immune checkpoints and virus infections [21, 22, 25, 127, 225].

One critical immune regulator that is regulated by UFMylation is the transcription factor NF-κB [226–228], which is known to induce pro-inflammatory genes in both innate and adaptive immune cells [229–231]. While the role of UFM1 in the context of NF-κB for neurons or in brain remains unclear, data from other cell types indicate complex and cell-type specific functions of UFM1. In human umbilical vein endothelial cells, UFM1 inhibits lipopolysaccharide (LPS) induced inflammation via NF-κB [232]. In goat endometrial epithelial cells, overexpression of UFM1 suppresses LPS-induced activation of the TLR4/NF-κB pathway [233]. Similarly, in human osteoarthritis chondrocytes, UFL1 has been shown to inhibit NF-κB activation triggered by IL-1β [234]. UFL1 was found to modulate NLRP3 inflammasome activation and protect against pyroptosis in LPS-stimulated bovine mammary epithelial cells. This protective effect was partly mediated through the inhibition of NF-κB signaling [235]. However, in the context of diabetes, UFMylation has been linked to enhanced inflammatory responses. UFM1 expression is elevated in macrophages from db/db (diabetic) mice and in LPS-stimulated macrophages, leading to increased pro-inflammatory cytokine production (e.g., TNF-α, IL-1β, IL-6) via activation of the NF-κB pathway through reduced LZAP expression [227]. Additionally, UFM1 promoted NF-κB p65 nuclear translocation in LPS-treated RAW264.7 cells and peritoneal macrophages in db/db mice by increasing the ubiquitination and degradation of IκBα [226]. In summary, the UFMylation pathway critically regulates the TLR4/NF-κB pathway, exerting a context- and cell-type-specific influence on inflammation by modulating NF-κB activation and pro-inflammatory cytokine production. However, its role in regulating the TLR4/NF-κB pathway during neuroinflammation remains unclear. In AD, activation of NF-κB is correlated with the production of pro-inflammatory cytokines, neuroinflammation, augmented Aβ production, and tau hyperphosphorylation, and neuronal death, which constitute key pathological hallmarks of the disease [17, 18, 212, 213]. A meta-analysis further identified NF-κB signaling as one of the most significantly disrupted pathways in late-onset AD brains [236]. In line with a role for pathogenesis, targeted inhibition of microglial NF-κB activation provided protection against Aβ toxicity in glial-neuron co-cultures [237]. TLR4 is also implicated in neuroinflammation in AD. Increased TLR4 expression has been observed in the brains of AD patients and familial AD mouse models [238]. In addition, single nucleotide polymorphisms (SNPs) in TLR4 have been associated with variations in AD risk [239, 240]. Hyperactivation of TLR4 has been shown to contribute to neuroinflammation in AD, with evidence indicating that Aβ can bind to TLR4, leading to immune activation and subsequent neuroinflammatory responses [238, 241–243]. Given the central role of the TLR4/NF-κB pathway in AD progression and the strong link between UFMylation and this signaling axis, targeting UFMylation alongside TLR4 and NF-κB signaling pathways may present novel therapeutic strategies to mitigate neuroinflammation and its detrimental effects in AD.

PD-1 and PD-L1 are key immune checkpoint molecules that regulate immune responses and are involved in the immune evasion of tumors [244, 245]. Both proteins can be UFMylated, but this modification has opposing effects depending on the context. In liver tumors, the UFMylation of PD-L1 facilitates its proteasome-mediated degradation, thereby suppressing tumor growth [246]. Conversely, UFL1 enhances the UFMylation of PD-1, stabilizing PD-1 and hindering the activation of CD8 + T cells, which promotes immune evasion [247]. In triple-negative breast cancer, UFM1 upregulates PD-L1 expression indirectly by stabilizing PLAC8, a protein highly expressed in this cancer type. This stabilization enhances PD-L1 expression through the modulation of its ubiquitination, contributing to tumor proliferation and immune modulation [248]. While a role of UFM1 for PD-L1 in the nervous system remains unknown, emerging evidence suggests that the PD-L1/PD-1 immune checkpoint pathway plays a critical role in modulating neuroinflammation, microglial function, amyloid-β clearance and p-tau clearance in AD [215, 216, 249]. Michal Schwartz’s team demonstrated that targeting PD-1 or its ligand PD-L1 with systemic injections of blocking antibodies could alter AD pathology in multiple mouse models. These include models of amyloid pathology (APP/PS1 and 5XFAD) and tau pathology (DM-hTAU, expressing human-tau with frontotemporal dementia mutations) [215, 216]. Blocking PD-1 signaling recruited monocyte-derived macrophages to the brain and facilitated the clearance of Aβ plaques and p-tau while improving cognitive performance. However, in contrast, a study by Kummer et al. found that PD-1 deficiency in APP/PS1 mice increased Aβ plaque accumulation and decreased microglial Aβ uptake, exacerbating neuroinflammation [249]. They also identified increased PD-L1 expression in astrocytes near Aβ plaques and higher PD-1 levels in microglia, suggesting a protective role for PD-1/PD-L1 signaling in AD. These findings highlight the dual role of PD-1/PD-L1 in AD, suggesting systemic PD-1 blockade may offer therapeutic potential, while local PD-1 deficiency might exacerbate neuroinflammation and disease severity. Whether changes in UFMylation alleviate or exacerbate microglial responses, Aβ clearance, and tau pathology by regulating PD-1/PD-L1 remains to be further explored.

Recent research has highlighted a crucial role of the UFM1 cascade for antiviral innate immunity [250, 251]. In the context of DNA virus infection, such as with HSV-1 or vaccinia virus (VACV), UFL1 protein levels are significantly reduced in peritoneal macrophages, suggesting involvement in promoting antiviral immunity. UFL1 was shown to regulate the cGAS-STING pathway by stabilizing STING protein, through competitive binding that prevents Lys48-linked ubiquitination, thereby enhancing antiviral responses [250], in a UFM1-independent fashion. It is interesting to note that HSV-1 has been associated with an increased risk of AD, especially in individuals carrying the APOE ɛ4 allele [218, 219] and it was shown that HSV-1 infection leads to the accumulation of Aβ and p-tau in cell cultures and 3D human brain models [252–254]. In RNA virus infections, UFL1 plays a critical role by dynamically relocating to mitochondrial-associated ER membranes, where it UFMylates mitochondrial antiviral signaling protein (MAVS), promoting its dislocation, incorporation into mitochondrial-derived vesicles (MDVs), and lysosomal degradation [255, 256]. This process is essential for RIG-I signaling, a key RNA virus sensor [251]. UFL1 also interacts with 14-3-3ε and RIG-I, leading to 14-3-3ε UFMylation, enhanced RIG-I activation, MAVS signaling, and interferon (IFN) production [251]. Additionally, UFL1-mediated UFMylation of RPL26 influences hepatitis A virus (HAV) RNA translation and replication [257]. While HAV infection has not been associated with AD, other RNA viruses, such as HCV and SARS-CoV-2, have been linked to AD [217, 225, 258, 259]. Furthermore, it has been shown that the level of the UFMylation substrate 14-3-3ε is significantly reduced in the frontal cortex of postmortem AD patients [260]. Investigating whether UFMylation plays a role in AD-related antiviral immunity could provide valuable insights. Further investigation is warranted to explore whether modulating UFMylation could offer a therapeutic avenue for reducing the risk of AD linked to viral infections.

The role of UFMylation for immune regulation is very complex and dynamic and the current body of evidence suggests that the UFM1 cascade is neither universally suppressive or promotive but rather tailored to the specific physiological or pathological context. Future studies are needed to elucidate the cell-type- and context-specific roles of UFMylation specifically for the nervous system and in the context of AD.

### Other pathways

In addition to the pathways previously mentioned, several studies have suggested that the UFMylation pathway may also be involved in other AD-related pathways, including:

#### Mitochondrial function

Mitochondrial impairment is a key feature of AD [261–263] and there are some links between the UFMylation pathway and mitochondrial function as well. Impairment in the UFMylation process leads to decreased mitochondrial density in aged brains via Atg9, mirroring the previously reported mitochondrial abnormalities observed in the indirect flight muscles of flies with Atg9 knockdown [184]. In addition, P4HB, the target for UFMylation that triggers ER stress also has mitochondrial effects. Ineffective UFMylation of P4HB leads to mitochondrial dysfunction and heightened oxidative stress [134]. Thus, UFMylation pathway may be linked to AD through its involvement in mitochondria function regulation.

#### Ribosome-associated protein quality control

Ribosome dysfunction is an early event in AD [264]. This impairment in ribosome function leads to reduced protein synthesis rate and capacity, a decline in ribosomal RNA and tRNA levels, and an increase in RNA oxidation [265, 266]. Certain ribosome proteins, such as uS3, uS10, and RPL26, have been recognized as targets of UFMylation [267]. Specifically, UFMylation of RPL26 increases during ribosome stalling, signaling translation errors and activating the ribosome-associated quality control mechanism during protein synthesis [267, 268]. Therefore, the UFMylation pathway may be connected to AD through its role in ribosome quality control.

#### Ferroptosis

Ferroptosis is an iron-dependent lipid peroxidation-driven programmed cell death and recent research has indicated a significant association between ferroptosis and AD [269–271]. However, the precise underlying mechanism remains elusive. SLC7A11, a key regulator of ferroptosis, is stabilized by UFM1 modification [272]. Knockdown of UFM1 reduces SLC7A11 expression by decreasing its protein stability without affecting transcription. Metformin induces ferroptosis by downregulating SLC7A11 protein stability through inhibition of its UFMylation [272]. Thus, the UFMylation pathway may be involved in the dysregulation of ferroptosis in AD.

## Conclusions and future perspectives

In this review, we have explored the many connections between AD and UFMylation, a critical but understudied ubiquitin-like post-translational modification. Given these many links, it is conceivable that the dysregulation of UFMylation could contribute to risk, onset or progression of AD and offer new therapeutic possibilities for intervention. A deeper understanding of the interplay between AD and UFMylation is needed to understand the multifaceted cellular processes and potential therapeutic outcomes. However, it is also important to note that dysfunctions in DDR, ER homeostasis, autophagy and immune responses are not exclusive to AD but are also prevalent in various other neurodegenerative disorders. These include Parkinson’s disease, Frontotemporal dementia, Amyotrophic Lateral Sclerosis, progressive multiple sclerosis and Huntington’s disease [119, 273–278]. This commonality suggests that deregulation of the UFMylation pathway could be a contributing factor in the pathogenesis of several of these neurodegenerative conditions.

One major shortfall in order to understand the role of UFM1 for neurological disease is that UFM1 function has not been sufficiently studied in brain. While there is a clear genetic link between UFMylation and neurodevelopmental disease, the majority of UFMylation research up to this point has focused mostly on non-neuronal cells. Neuronal effects remain understudied and nervous system-specific targets of UFM1 have not been identified yet, Therefore, it will be important to shift the focus and intensify research on brain-centric cells, including neurons, microglia, oligodendrocytes, and astrocytes. Such studies are essential to unravel the physiological and potentially pathological roles of UFMylation in the nervous system, which will be pivotal to understand the role of UFM1 plays for neuronal biology and its pathological impact on conditions such as neurodevelopmental and neurodegenerative diseases.

Given that the reduction of UFMylation has been shown to reduce the propagation of tau induced by seeding in vitro and in vivo [61], it is conceivable that UFM1 plays an important role for the pathogenesis of tauopathies. It will be important to shed light onto the mechanism of how UFM1 influences tau. It has recently been shown that the UFM1 cascade also regulates α-synuclein levels [279] and it was shown that mono-UFMylation of α-synuclein modulates its secretion, which then affects the spreading of aggregated synuclein. It remains to be seen whether a similar mechanism could also account for other misfolded proteins, such as tau, as suggested by the authors [280]. More research into the molecular mechanism and exploring the impact of UFMylation on biological and pathological levels of tau (Aβ, and α-synuclein), remains paramount. Understanding how UFMylation modifies these proteins and affects their aggregation, toxicity, clearance, and transmission will provide critical insights into the pathogenesis and progression of neurodegenerative diseases. It is currently unclear whether UFM1 is directly conjugated to tau in neurons or in brain. A thorough characterization of the UFMylation proteome in neuronal cells and human brain specimens is needed to identify specific substrates exhibiting increased UFMylation in AD-affected brains. These substrates may play a critical role in the development or progression of AD - offering a clearer understanding of the disease at a molecular level; might provide new biomarkers for early detection, monitoring, and prognosis; and help to identify tractable therapeutic targets for people AD.

New tools are needed to understand UFMylation on the molecular level. This includes high-quality antibodies, relevant animal models, gene edited human stem cell derived neurons, and pharmacological compounds to modulate this pathway, including antagonists and agonists. Since UFMylation involves a set of UFM1-specific enzymes that catalyze its addition and deconjugation, such compounds should be highly specific for just UFM1 and not affect other UBL modifications such as SUMOylation, NEDDylation or ISG15ylation. These tools will be important to pave the way for a more profound exploration of the UFMylation pathway, its druggability and therapeutic potential. While the molecular intricacies connecting AD, neurodevelopmental disorders, and UFMylation remain to be fully elucidated, we advocate for intensified research endeavors aimed at pinpointing UFMylation targets in these diseases.

In conclusion, while there has been limited exploration into the role of UFMylation in neurodegenerative diseases, the emerging research, especially in the context of AD, is promising. Future in-depth studies in this domain have the potential to not only deepen our understanding but also pave the way for innovative therapeutic strategies for affected individuals. Dissecting the molecular functions of UFMylation especially with regards to AD might offer new and exciting opportunities for disease intervention.

## Data Availability

No datasets were generated or analysed during the current study.

## Abbreviations

AD: Alzheimer’s disease
Aβ: Beta-amyloid
APP: Amyloid precursor protein
DDR: DNA damage response
DSBs: Double-strand breaks
ER: Endoplasmic reticulum
HAV: Hepatitis a virus
HR: Homologous recombination
HSV-1: Herpes simplex virus
IFN: Interferon
iPSC: Induced pluripotent stem cell
KO: Knock out
LPS: Lipopolysaccharide
MAVS: Mitochondrial antiviral signaling protein
MDVs: Mitochondrial-derived vesicles
NHEJ: Non-homologous end joining
p-ATM: Phospho-ser1981 ATM
ROS: Reactive oxygen species
SARS‑CoV‑2: Severe acute respiratory syndrome coronavirus 2
SNPs: Single nucleotide polymorphisms
TNF: Tumor necrosis factor
UBL: Ubiquitin-like
UPR: Unfolded protein response
VACV: Vaccinia virus
